# Prevalence and factors associated with fear of COVID-19 in military personnel during the second epidemic wave in Peru

**DOI:** 10.3389/fpsyt.2024.1309957

**Published:** 2024-03-07

**Authors:** Danai Valladares-Garrido, Helena Dominguez-Troncos, Cinthia Karina Picón-Reátegui, Christopher Valdiviezo-Morales, Víctor J. Vera-Ponce, Virgilio E. Failoc-Rojas, César Johan Pereira-Victorio, Darwin A. León-Figueroa, Mario J. Valladares-Garrido

**Affiliations:** ^1^ Facultad de Medicina, Universidad Cesar Vallejo, Trujillo, Peru; ^2^ School of Medicine, Universidad Nacional de Piura, Piura, Peru; ^3^ Scientific Society of Medical Students from Universidad Nacional de Piura, Piura, Peru; ^4^ Facultad de Medicina Humana, Universidad de San Martín de Porres, Chiclayo, Peru; ^5^ Facultad de Medicina, Universidad Nacional Mayor de San Marcos, Lima, Peru; ^6^ Instituto de Investigación de Enfermedades Tropicales, Universidad Nacional Toribio Rodríguez de Mendoza de Amazonas, Chachapoyas, Amazonas, Peru; ^7^ Universidad Tecnológica del Perú, Lima, Peru; ^8^ Research Unit for Generation and Synthesis Evidence in Health, Universidad San Ignacio de Loyola, Lima, Peru; ^9^ School of Medicine, Universidad Continental, Lima, Peru; ^10^ Facultad de Medicina, Universidad Cesar Vallejo, Piura, Peru; ^11^ Oficina de Epidemiología, Hospital Regional Lambayeque, Chiclayo, Peru

**Keywords:** COVID-19, fear, mental health, military, Peru

## Abstract

**Introduction:**

During the COVID-19 pandemic, the mounting workload and heightened stress may contribute to exacerbated mental health challenges, including an increased fear of COVID-19, among military personnel. Despite the potential influence of these factors, there remains a scarcity of studies addressing mental health issues, particularly the fear of COVID-19, within this specific population. We aimed to determine the prevalence and factors associated with fear of COVID-19 among military members.

**Methods:**

A cross-sectional study was conducted between November 2 and 9, 2021, during the second wave of the COVID-19 pandemic in the Lambayeque region, Peru. The outcome variable was fear of COVID-19, assessed using the Fear of COVID-19 Scale. The association with resilience (Connor-Davidson Resilience Scale, abbreviated as CD-RISC), food insecurity (Household Food Insecurity Access Scale, abbreviated as HFIAS), physical activity (International Physical Activity Questionnaire-Short Form, abbreviated as IPAQ-S), eating disorder (Eating Attitudes Test-26, abbreviated as EAT-26), and other socio- demographic variables was assessed.

**Results:**

Among the 525 participants, the median age was 22, 95.8% were male, and 19.2% experienced fear of COVID-19. A higher prevalence of fear of COVID-19 was associated with age (PR=1.03; 95% CI: 1.01-1.06), religion (PR=2.05; 95% CI: 1.04-4.05), eating disorder (PR=2.95; 95% CI: 1.99-4.36), and having a relative with mental disorder (PR=2.13; 95% CI: 1.09-4.17). Overweight (PR=0.58; 95% CI: 0.37-0.90) and a high level of resilience (PR=0.63; 95% CI: 0.43-0.93) were associated with a lower prevalence of fear of COVID-19.

**Discussion:**

Two out of ten military personnel were afraid of COVID-19. Our results highlight the need for targeted interventions addressing the factors contributing to fear of COVID-19 among military personnel, emphasizing the significance of mental health support and preventive measures within this specific population.

## Introduction

Since the onset of the COVID-19 pandemic, it has posed a threat to both physical (1–4) and psychological health (5–14). This impact, coupled with its effects on social and economic aspects, has had far-reaching consequences on the safety and overall well-being of the population (15, 16). The severity (2, 17) and high transmission rate of COVID-19 (18, 19) have instilled fear in people (15, 20). This fear is exacerbated by misinformation, leading to the psychological impact of concerns about infection and the potential to infect loved ones (20, 21).

The estimated prevalence of fear of COVID-19 varies between 30.5% and 41.8% in different populations (22, 23). Notably, a study conducted among Peruvian policemen reported an even higher rate, with 42.5% expressing fear of COVID-19 (24). In Latin America, an average fear percentage of 15.54 has been described on the COVID-19 fear scale (25), which is comparatively lower than other continents, ranging from 21.7% to 23.8% (23).

While fear of COVID-19 has been extensively studied in healthcare personnel (26), there is a notable gap in research concerning military personnel, despite their crucial role in the fight against COVID-19 (5, 27). This population bears the responsibility of providing protection and security to citizens, often involving tasks such as setting up temporary hospitals, ensuring compliance with preventive measures, and assisting in the transfer of patients or handling of deceased individuals (28). Consequently, military personnel could be exposed to traumatic experiences, risking both their psychological and physical well-being, similar to healthcare personnel (29). A study conducted in the Spanish Armed Forces revealed that 52.6% of participants felt the need for psychological help in anticipation of a new wave of the pandemic, and 49.2% reported experiencing fear of death (28).

Studies in the general population have found factors associated with fear of COVID-19 such as being aged between 50 and 64 years (30), being a female (31), low resilience, having chronic diseases (31, 32), anxiety (33), alcoholism (31), smoking (31), perception of getting infected with COVID-19, and fear of risk for loved ones (33). However, the instrument used to measure fear of COVID-19 was not validated (30), nor did the authors investigate other variables of interest such as religious aspects, eating disorders or family history of mental health, which we have evaluated in a military population.

In the landscape of the COVID-19 pandemic, the psychological well-being of military personnel emerges as an essential yet often overlooked component. Men and women in active service, occupying crucial roles in the pandemic response, face a unique amalgamation of challenges and tensions. The constant exposure to risks during operations, the emotional burden of protecting the population, and the uncertainty associated with the nature of the pandemic shape a uniquely stressful environment (34).

The justification for conducting this study is grounded in the urgent need to comprehensively understand how the fear of COVID-19 permeates the mental health of military personnel (5, 35). This group, essential in pandemic management, faces particular pressures and fears. By exploring underlying factors, this study aims to expand existing knowledge and provide fundamental insights that have so far remained in the shadows of research.

The uniqueness of the military context, characterized by constant duty and exposure to extraordinary situations, highlights the need for a precise and comprehensive approach in researching the fear of COVID-19. The results derived from this study will not only contribute to the scientific foundation related to mental health in crisis situations but will also inform specific strategies and policies that effectively address psychological concerns in this specialized group.

This study seeks to address this gap by delving into the prevalence and factors associated with fear of COVID-19 among Peruvian military personnel during the second wave of the pandemic. Military personnel, integral to enforcing preventive measures, ensuring public safety, and contributing to patient care, face unique challenges that expose them to potential trauma and psychological stress (28, 29). Existing studies within the military context, such as those in the Spanish Armed Forces, underscore the need to explore the psychological well-being of military personnel during the ongoing pandemic (28).

Our research not only contributes to the broader understanding of fear of COVID-19 but also expands the scientific evidence by examining factors such as age, religiosity, body weight, family history of mental health, and eating disorders in this specific population. Religious aspects are considered, acknowledging their potential influence on coping mechanisms and mental well-being, especially in the high-stress military environment where individuals may turn to their beliefs for support and resilience (36–38). The study delves into eating disorders, recognizing the complex relationship between mental health, stress, and unique eating patterns that may emerge in response to the specific stressors faced by military personnel (22, 39). Additionally, exploring the family history of mental health aims to provide insights into individual susceptibility or resilience to stressors, including those associated with the COVID-19 pandemic, within the military context (40, 41).

Furthermore, our study highlights the significance of resilience as a distinctive protective factor in understanding the fear of the virus among military personnel (discussed in the discussion section). By uncovering these nuanced associations, we aim to provide essential insights that inform tailored strategies, policies, and interventions to address the psychological impact on military personnel—insights that are crucial in navigating the complexities of mental health during crises. Through a comprehensive exploration of fear of COVID-19 in military personnel, this study endeavors to pave the way for evidence-based psychological interventions that cater specifically to the unique challenges faced by this essential group in the context of the post-pandemic world and future health emergencies or disasters.

Given the importance of understanding the psychological impact of the COVID-19 pandemic on military personnel, this study seeks to contribute valuable insights into the prevalence and factors associated with fear of COVID-19 in this specific population. It is essential to clarify that while this research shares a common participant pool and data collection duration with a larger study (42), the focus and objectives of this investigation are distinct and represent an independent analysis. This ensures transparency and acknowledges the connection with previous research, avoiding any ambiguity regarding the nature of this study. While the present study shares participants and data collection duration with a larger primary investigation (42), it is crucial to emphasize that this work represents a distinct secondary analysis. The primary study aimed at determining the prevalence and factors associated with depression and anxiety (42). In this secondary analysis, we specifically focus on unraveling the prevalence and factors associated with fear of COVID-19 among military personnel. This approach ensures transparency and elucidates the relationship between the two studies, underscoring the unique contribution of this research in addressing the psychological implications of the pandemic on military personnel.

Our research aims to expand the scientific evidence on fear of COVID-19 in military personnel to know the psychological impact that the pandemic has caused in this population and to allow the future implementation of psychological interventions to cope with this fear. Therefore, this study aims to address the following questions: What is the prevalence of fear of COVID-19 among Peruvian military personnel during the second pandemic wave, and what factors are associated with this fear in this specific population?

## Materials and methods

### Study design and population

This study is analytical and cross-sectional. We used a secondary data analysis to identify the prevalence and the factors associated with fear of COVID-19 in military personnel in Lambayeque, Peru. The primary study aimed to assess the prevalence and factors associated with depressive and anxious symptoms in military personnel (42).

The population of the study is comprised of 820 military personnel in charge of the first line of defense of the health emergency due to COVID-19 in the city of Lambayeque, Peru.

By the primary study, we aimed for a sample size of 582 military personnel, considering a precision of 2.5%, a confidence level of 99%, an expected prevalence of 12.8%, and accounting for a 20% allowance for potential missing data or refusals. During the study, we successfully achieved the participation of 86.6% (n = 710) of the population, exceeding our initial expectations (42).

In the primary study, the military personnel that were included were those who were working for one month, at least, at the moment of the administration of the survey. Exclusions included individuals who declined to provide consent for study participation (n=10), those working remotely due to a high risk of COVID-19 (n=6), individuals in quarantine due to confirmed coronavirus infection (n=5), and those who had been working for less than one month (n=8). Additionally, 81 individuals could not be invited as they were not present during the data collection period. Furthermore, we excluded 95 military personnel who did not correctly complete the instruments related to depression and anxiety. Therefore, the final sample used for the primary study analysis comprised 615 individuals (42). The sample selection for this study involved several considerations. The initial access to the internet was facilitated through the smartphones owned by each military participant. In cases where individuals did not have internet access, data-sharing arrangements were made for their participation. All participants were proficient in reading and writing in Spanish, as the study involved Peruvian military personnel.

For this research, we excluded 90 records of military personnel who did not complete the Fear COVID-19 Scale. This scale measured the outcome variable of this research. Therefore, the sample selected for this secondary data analysis consisted of 525 military personnel. The response rate for the study was 64.02% (525/820). Regarding the 525 military personnel selected for this analysis, we found that the median age was 22, 95.8% were male, 17.1% consumed alcohol frequently, and 8.2% reported having sought mental health support due the COVID-19 pandemic.

Additionally, since the study focused on a specific population with unique characteristics and faced restrictions due to the pandemic and social distancing measures, snowball sampling was employed to reach military personnel more effectively. This method was chosen to ensure the inclusion of participants who might be harder to reach through conventional sampling approaches.

For this secondary analysis, the statistical power of the eating disorder and resilience factors was estimated. In the case of the eating disorder, a statistical power value of 99.27% was estimated. This calculation was based on the fear of COVID-19 proportion in the group without an eating disorder (p1 = 0.163) and the corresponding proportion in the group with an eating disorder (p2 = 0.444). Additionally, the respective sample sizes were considered, with n1 = 471 for the group without an eating disorder and n2 = 54 for the group with an eating disorder. Regarding resilience, a statistical power value of 77.15% was estimated. This calculation was based on the fear of COVID-19 proportion in the low resilience group (p1 = 0.233) and the corresponding proportion in the high resilience group (p2 = 0.140). Additionally, the respective sample sizes were considered, with n1 = 296 for the low resilience group and n2 = 229 for the high resilience group.

The study adhered to the STROBE (Strengthening the Reporting of Observational Studies in Epidemiology) cross-sectional reporting guidelines to ensure comprehensive and transparent reporting of the research methods and findings.

### Procedure

Authorization was requested from the infantry brigade of Lambayeque to conduct the research with military personnel. Data collection forms were created using the REDCap system to ensure optimal data quality control, and an online questionnaire link was generated. However, it is important to clarify that all data, including responses to the questionnaire, was collected through self-administration by participants via the online questionnaire link, which was distributed to their smartphones while they were gathered in the military facility’s meeting room. Military personnel were gathered in groups, and the online questionnaire link was shared with them to access via their smartphones and respond to the research questions. The execution took place in person, adhering to strict biosecurity measures, including the mandatory use of masks, continuous hand sanitization, and utilization of open spaces for ventilation. Data collection occurred from November 2 to 9, 2021, organized in two shifts (morning and afternoon), and supervised by a military member of the 7th Infantry Brigade, located in the Lambayeque region, Peru. The questionnaire link was distributed to the study population through text messages, WhatsApp virtual messages, and internal coordination groups. Before initiating the questionnaire, participants were electronically asked for their informed consent to participate in the study.

To address potential issues like duplications or fraud in the online survey, we employed various strategies. Initially, participants were required to provide informed consent electronically before accessing the questionnaire, ensuring that only authorized individuals took part. The survey link was distributed through military channels, and participants were continuously supported and overseen by their superiors or military officers during execution. This ensured that all respondents were indeed military personnel.

Additionally, we utilized the REDCap system, an advanced data entry tool. Within REDCap, we implemented several measures to ensure the correct arrangement, provision, and completion of questionnaires. These measures included unique and anonymized identifiers on each form, consistent questionnaire ordering (beginning with consent and informed assent, followed by general data and variables of interest instruments), conditional logic for skip questions, mandatory fields to prevent missing information, and minimum and maximum ranges in numerical variables. Moreover, we incorporated the REDCapcha feature to detect and deter traffic from automated programs or bots.

### Variable and measures

#### Dependent variable

##### Fear of COVID

The outcome variable, fear of COVID-19, was operationally defined as a score higher than 16.5, calculated by summing the responses to the 7 items of the Fear of COVID-19 Scale (43). This cutoff point has been validated in similar studies conducted on frontline COVID healthcare professionals (44) and the general population (43).

#### Independent variables

##### Food insecurity

it was assessed using the Household Food Insecurity Access Scale (HFIAS) based on the FANTA-III criteria for evaluation. Mild food insecurity (FI) is assigned with a score of 2 to 3 points in the first item, 1 to 3 in the second item, or 1 in the third or fourth item. The scoring for moderate FI includes 2 to 3 points in the third or fourth item, or 1 to 2 in the fifth or sixth item. Severe FI is characterized by a score of 3 points in the fifth or sixth item, or 1 out of 3 in the seventh, eighth, or ninth item. Finally, the variable was dichotomized into the absence of food insecurity and the presence of food insecurity, including mild, moderate, and severe levels.

##### Physical activity

This was assessed using the International Physical Activity Questionnaire IPAQ-S (short version). Low physical activity was operationally defined as engaging in less than 150 minutes per week of moderate physical activity. Moderate physical activity was defined as any activity lasting at least 10 minutes and requiring moderate physical effort. Vigorous physical activity was defined as any activity performed for at least 10 minutes and requiring intense physical effort.

##### Eating disorder

It was obtained with the EAT-26, Eating Attitudes Test, after categorizing the score obtained about the absence or presence of such disorder, using a cut-off point of 20 points.

##### Resilience

It is operationally defined as a score above 30 points obtained from the answers of the Connor-Davidson questionnaire in the abbreviated version (45).

##### Socio-demographic variables

Among them, we can mention age in years, gender (female, male), marital status (single, married, cohabiting, divorced), religion (none, Catholic, non-Catholic), having children (no, yes), frequent consumption of alcohol (no, yes) and tobacco (no, yes), body mass index (underweight, normal, overweight, obese), previous history of mental health (no, yes), previous family history of mental health (no, yes), report of having sought mental health support (no, yes), confidence in the government to handle the pandemic (no, yes), and how long the sample was working during the COVID-19 health emergency (one to six months, seven to twelve months, thirteen to eighteen months, nineteen months, or more).

**Figure 1:**
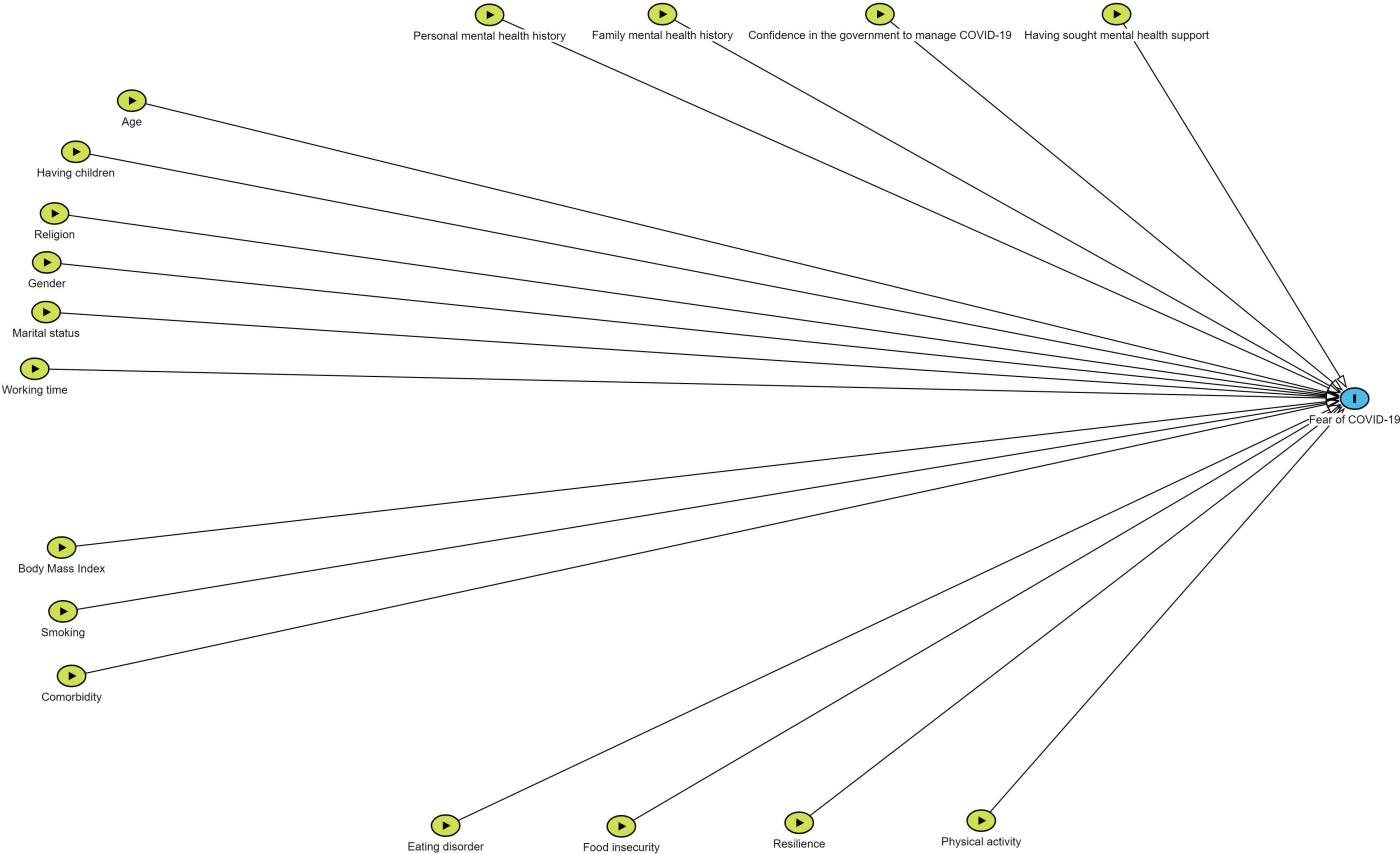
Directed Acyclic Graph (DAG) of the factors associated with fear of COVID-19.

**Figure 1** shows a Directed Acyclic Graph (DAG) illustrating the influence of confounding variables **Figure 1**.

#### Measures

##### Fear of COVID scale

This scale consists of seven items and is reliable and valid for assessing fear of COVID-19 among the general population, with a Cronbach’s alpha of 0.82 (46). An investigation of the psychometric properties of the Spanish version of the Fear of COVID-19 Scale in a sample of the Peruvian population showed that this brief scale of fear of COVID-19 has adequate measurement properties both in terms of reliability and validity (47).

##### Household food insecurity access scale

It is composed of nine questions that assesses food access of the last four weeks. It has adequate internal consistency (Cronbach’s alpha of 0.74) (48). Also, this scale has the following categories: food security, mild, moderate and severe food insecurity (49).

##### International physical activity questionnaire - short form

This instrument is composed of seven questions that evaluate physical activity in the last week. It is divided into low, moderate and high physical activity, after a weighted estimate of total physical activity reported in the last week (50). It shows adequate psychometric properties in Latin American population (50–52). In its abbreviated version, adequate correlations (0.26-0.69) in Spanish-speaking population have been estimated (53).

##### Eating attitudes test-26 item

This instrument is made up of 26 items measured with a Likert scale (never, rarely, sometimes, often, very often and always). It has been validated in male population from Colombia (Cronbach’s alpha 0.98, sensitivity: 100%, specificity: 97.8%) (54) It has been validated in the female population of Latin America (55, 56), demonstrating adequate reliability (Cronbach’s alpha: 0.92; sensitivity of 100% and specificity of 86.6%) in women from Colombia (56).

##### Connor Davidson abbreviated questionnaire

This questionnaire is composed of ten questions and has excellent psychometric properties (Cronbach’s alpha of 0.89) in the general population (57).

### Statistical analysis

#### The statistical analysis was conducted using Stata 17

In the descriptive analysis, we calculated frequencies and percentages for categorical variables. For the numerical variable, age, which exhibited a non-normal distribution, we reported the median and interquartile range.

For hypothesis testing, we employed the Chi-squared test to assess the association between categorical independent variables and the outcome variable (fear of COVID). Regarding age, a numerical variable, we applied the Mann-Whitney U test after confirming the non-normal distribution assumption.

To delve into the factors associated with fear of COVID, we constructed both simple and multiple regression models. Prevalence ratios (PR) with corresponding 95% confidence intervals (95% CI) were estimated using generalized linear models with a Poisson distribution family and a log-link function, incorporating robust variance.

### Ethical aspects

The primary study was ethically approved by the Ethics Committee of Universidad San Martín de Porres (USMP) with the code 269-2022-CIEI-FMH-USMP. Participants were fully informed about the voluntary nature of their participation, and their privacy was strictly respected. Informed consent was obtained electronically from all participants, who were military personnel. No incentives were offered for participation, ensuring that involvement in the study was solely based on voluntary contributions. The collected data were treated with utmost confidentiality, and measures were in place to ensure anonymity.

## Results

Almost half of them had food insecurity (49.5%) and 43.6% had a high level of resilience. Also, 19.2% felt fear of COVID-19 **Table 1**.

**Table 1:** Characteristics of military personnel from Lambayeque, Peru (n=525).

Characteristics	N (%)
Age*	22 (19–31)
Gender	
Female	22(4.2)
Male	503(95.8)
Marital status	
Single	390(74.3)
Married	117(22.3)
Domestic partner	12(2.3)
Divorced	6(1.1)
Religion	
None	80(15.2)
Catholic	359(68.4)
Non-Catholic	86(16.4)
Having children	139(26.5)
Alcoholism	90(17.1)
Smoking	35(6.7)
Comorbidity	
Hypertension*	50(9.5)
Diabetes	10(1.9)
BMI (categorized)**	
underweight/normal	312(60.4)
Overweight	172(33.3)
Obesity	33(6.4)
Personal mental health history	
No	518(98.7)
Yes	7(1.3)
Family mental health history	
No	502(95.6)
Yes	23(4.4)
Having sought mental health support	
No	482(91.8)
Yes	43(8.2)
Confidence in the government to manage COVID-19	
Yes	288(54.9)
No	237(45.1)
How long have you been working?**	
1 to 6 months	134(26.2)
7 to 12 months	82(16.0)
13 to 18 months	110(21.5)
19 months or more	186(36.3)
Food insecurity	
No	265(50.5)
Yes	260(49.5)
Physical activity	
Low	64(12.2)
moderate	37(7.1)
High	424(80.8)
Eating disorder	
No	471(89.7)
Yes	54(10.3)
Resilience	
low	296(56.4)
high	229(43.6)
Fear of COVID-19	
No	424(80.8)
Yes	101(19.2)

* Median (25th percentile - 75th percentile).

**Missing values.

**Figure 2:**
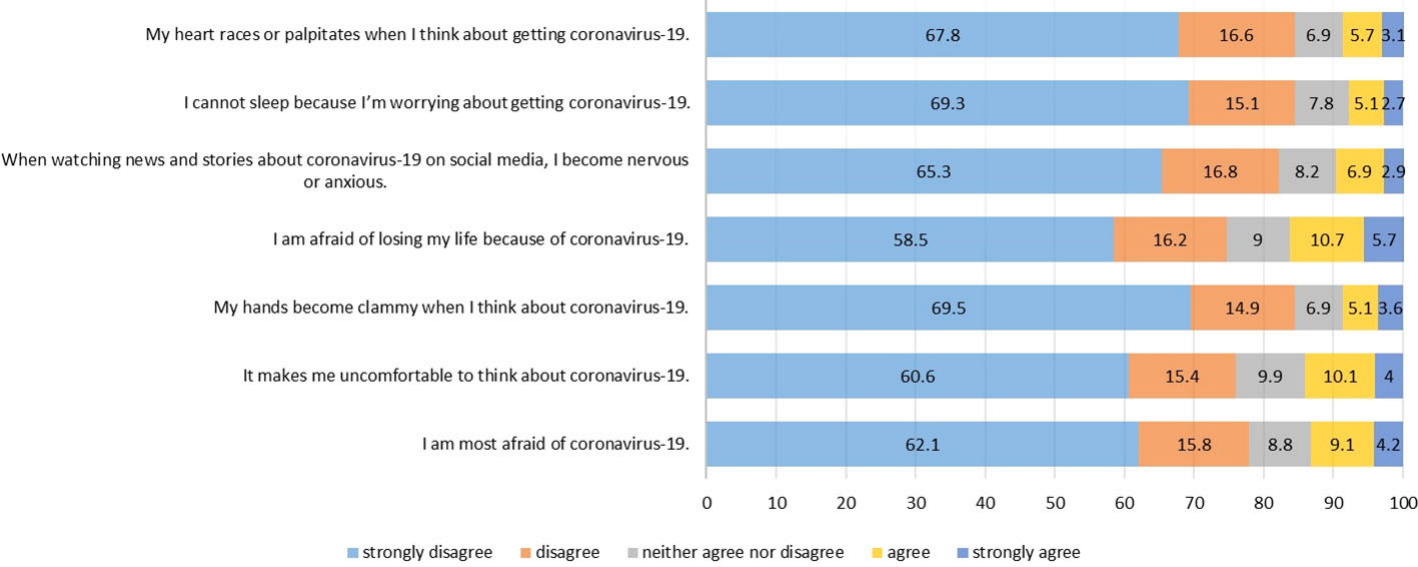
Fear of COVID-19 Scale.

A total of 10.7% and 10.1% mentioned having felt fear of losing their lives due to coronavirus and having felt uncomfortable thinking about coronavirus, respectively. In addition, 9.1% reported having felt much fear of COVID-19 **Figure 2**.

We found that the participants with a low level of resilience had a higher level of prevalence of fear of COVID, in comparison with those who had a high level of resilience (23% vs. 14%; *p*=.007). Moreover, military personnel with eating disorders showed a higher prevalence of fear of COVID, in comparison with those who did not have that disorder (44.4% vs. 16.4%; *p*<.001). Furthermore, age in years, religion, frequent consumption of alcohol and tobacco, having a relative with mental illnesses, and how long they have been working were associated with fear of COVID-19 **Table 2**.

**Table 2:** Factors associated with fear of COVID-19 in military personnel, bivariate analysis.

Variables	*Fear of COVID*		p*
	No (n=424) n(%)	Yes (n=101) n(%)	
Age (years)***	21(19-31)	25(20-36)	0.004**
Gender			0.329
Female	16(72.7)	6(27.3)	
Male	408(81.1)	95(18.9)	
Marital status			0.312
Single	321(82.3)	69(17.7)	
Married	91(77.8)	26(22.2)	
Domestic partner	8(66.7)	4(33.3)	
Divorced	4(66.7)	2(33.3)	
Religion			**0.035**
None	72(90.0)	8(10.0)	
Catholic	288(80.2)	71(19.8)	
Non-Catholic	64(74.4)	22(25.6)	
Having children	106(76.3)	33(23.7)	0.116
Alcoholism	62(68.9)	28(31.1)	**0.002**
Smoking	20(57.1)	15(42.9)	**<0.001**
Comorbidity			
Hypertension*	38(76.0)	12(24.0)	0.369
Diabetes	7(70.0)	3(30.0)	0.383
BMI (categorized)			0.993
underweight/normal	253(81.1)	59(18.9)	
Overweight	140(81.4)	32(18.6)	
Obesity	27(81.8)	6(18.2)	
Personal mental health history			0.110
No	420(81.1)	98(18.9)	
Yes	4(57.1)	3(42.9)	
Family mental health history			**0.013**
No	410(81.7)	92(18.3)	
Yes	14(60.9)	9(39.1)	
Having sought mental health support			0.271
No	392(81.3)	90(18.7)	
Yes	32(74.4)	11(25.6)	
Confidence in the government to manage COVID-19			0.154
Yes	239(83.0)	49(17.0)	
No	185(78.1)	52(21.9)	
How long have you been working?			**0.015**
1 to 6 months	113(84.3)	21(15.7)	
7 to 12 months	64(78.1)	18(22.0)	
13 to 18 months	78(70.9)	32(29.1)	
19 months or more	158(85.0)	28(15.1)	
Food insecurity			0.828
No	215(81.1)	50(18.9)	
Yes	209(80.4)	51(19.6)	
Physical activity			0.121
Low	46(71.9)	18(28.1)	
moderate	32(86.5)	5(13.5)	
High	346(81.6)	78(18.4)	
Eating disorder			**<0.001**
No	394(83.7)	77(16.4)	
Yes	30(55.6)	24(44.4)	
Resilience			**0.007**
low	227(76.7)	69(23.3)	
high	197(86.0)	32(14.0)	

*P-value of categorical variables calculated with the Chi-squared test.

**P-value of categorical-numerical variables calculated with the U test (Mann-Whitney).

***Median - interquartile range.Bold values highlight statistically significant p-values associated with the variables.

**Figure 3:**
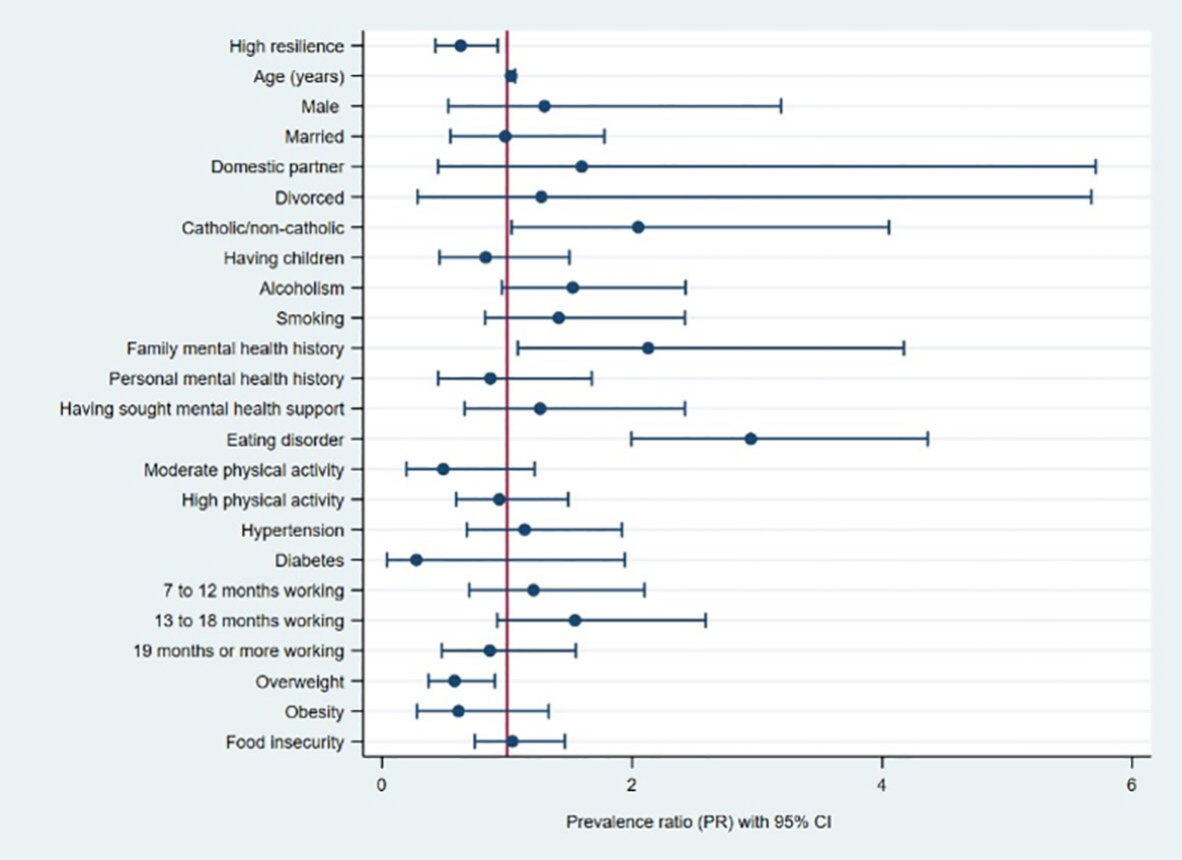
Forest plot of the factors associated with fear of COVID-19 in military personnel.

In the multiple regression model, we found that the factors associated with a high level of prevalence of fear of COVID-19 were age (in years) (PR=1.03; 95% CI: 1.01-1.06), having a religion (PR=2.05; 95% CI: 1.04-4.05), having a relative with a mental disorder (PR=2.13; 95% CI: 1.09-4.17) and having an eating disorder (PR=2.95; 95% CI: 1.99-4.36). In contrast, being overweight and having a high level of resilience reduces the prevalence of fear of COVID-19 in military personnel by 42% (PR=0.58; 95% CI: 0.37-0.90) and 37% (PR=0.63; 95% CI: 0.43-0.93) (**Table 3**, **Figure 3**).

**Table 3:** Factors associated with fear of COVID-19 in military personnel, simple and multiple regression analysis.

Characteristics	*Fear of COVID-19*					
	Simple regression			Multiple regression		
	PR	95% CI	p*	PR	95% CI	p*
Age (years)***	1.02	1.01-1.04	**0.003**	1.03	1.01-1.06	**0.041**
Gender						
Female	Ref.			Ref.		
Male	0.69	0.34-1.40	0.308	1.30	0.53-3.19	0.569
Marital status						
Single	Ref.			Ref.		
Married	1.26	0.84-1.88	0.266	0.99	0.55-1.78	0.962
Domestic partner	1.88	0.82-4.32	0.134	1.60	0.45-5.71	0.472
Divorced	1.88	0.59-5.97	0.281	1.27	0.29-5.67	0.751
Religion						
None	Ref.			Ref.		
Catholic/Non-Catholic	2.09	1.06-4.14	**0.034**	2.05	1.04-4.05	**0.039**
Having children	1.35	0.93-1.95	0.112	0.83	0.46-1.50	0.536
Alcoholism	1.85	1.28-2.69	**0.001**	1.52	0.96-2.42	0.075
Smoking	2.44	1.41-4.23	**0.001**	1.41	0.82-2.42	0.209
Comorbidity						
Hypertension*	1.28	0.86-2.17	0.358	1.14	0.68-1.92	0.125
Diabetes	1.58	0.60-4.14	0.355	0.28	0.04-1.94	0.787
BMI (categorized)						
underweight/normal	Ref.			Ref.		
Overweight	0.98	0.67-1.45	0.934	0.58	0.37-0.90	**0.016**
Obesity	0.96	0.45-2.06	0.919	0.61	0.28-1.33	0.216
Personal mental health history						
No	Ref.			Ref.		
Yes	2.27	0.95-5.43	0.067	0.87	0.45-1.68	0.671
Family mental health history						
No	Ref.			Ref.		
Yes	2.14	1.24-3.67	**0.006**	2.13	1.09-4.17	**0.028**
Having sought mental health support						
No	Ref.			Ref.		
Yes	1.37	0.80-2.36	0.256	1.26	0.66-2.42	0.480
Confidence in the government to manage COVID-19						
Yes	Ref.			Ref.		
No	1.29	0.91-1.83	0.155	1.05	0.72-1.53	0.781
How long have you been working?						
1 to 6 months	Ref.			Ref.		
7 to 12 months	1.40	0.79-2.47	0.244	1.21	0.70-2.10	0.497
13 to 18 months	1.86	1.14-3.03	**0.013**	1.54	0.92-2.59	0.100
19 months or more	0.96	0.57-1.62	0.880	0.86	0.48-1.55	0.620
Food insecurity						
No	Ref.			Ref.		
Yes	1.04	0.73-1.48	0.828	1.04	0.74-1.46	0.815
Physical activity						
Low	Ref.			Ref.		
moderate	0.48	0.19-1.19	0.113	0.49	0.20-1.22	0.125
High	0.65	0.42-1.02	0.059	0.94	0.59-1.49	0.787
Eating disorder						
No	Ref.			Ref.		
Yes	2.72	1.89-3.90	**<0.001**	2.95	1.99-4.36	**<0.001**
Resilience						
low	Ref.			Ref.		
high	0.60	0.41-0.88	**0.009**	0.63	0.43-0.93	**0.019**

*P-values obtained with Generalized Linear Models (GLM), Poisson family, log-link function, robust variance.Bold values highlight statistically significant p-values associated with the variables.

## Discussion

### Main findings

It was found that two out of every ten participants were afraid of COVID-19.

Higher prevalence of fear was associated with older age, religious affiliation, a family history of mental illness, and the presence of an eating disorder. Conversely, lower prevalence of fear was linked to being overweight and exhibiting a high level of resilience.

### Prevalence of fear of COVID-19

The prevalence of fear of COVID-19 in our study was found to be 19.2%, which contrasts with the higher incidence reported in a study by Caycho et al. in our country (42.5%) (24) and in Spain by Lázaro-Pérez et al. (80%) (28). The difference in prevalence could be attributed to the timing of the investigations. Caycho et al. conducted their study in 2020, during the peak of COVID-19 cases and deaths in our country (24), where the armed and police forces played a crucial role in enforcing security measures. Similarly, Lázaro-Pérez et al. conducted their research at the beginning of the second wave (August-September 2020), a period marked by the discovery of new virus variants and the announcement of a new confinement, leading to heightened anxiety in the Spanish population (28).

Despite the potential overall decrease in fear compared to earlier stages, military personnel faced unique challenges and stressors associated with their crucial roles in the pandemic response. Our study aims to provide valuable insights into the psychological impact on military personnel during this specific phase, contributing to a nuanced understanding of fear even when it might be relatively lower in the general population. Furthermore, we acknowledge that the incidence of fear of COVID-19 in our study might have been influenced by the timing of our investigations. However, the continuation of significant indicators of lethality and SARS-CoV-2 positivity during the second wave in Peru (58, 59), particularly in the Lambayeque region, necessitated the maintenance of strict measures, including social distancing, mandatory social isolation, and targeted quarantines. Frontline personnel, including the military, played a crucial role in ensuring compliance with these measures.

### Resilience and fear of COVID-19

Participants with a high level of resilience had a 47% lower prevalence of fear of COVID-19. This finding coincides with what was reported by Satici et. al, who found that during the first months of the pandemic people with resilience felt less fear of COVID-19 because of a mechanism involving subjective hope and happiness in the face of adversity (60). It aligns with findings reported in Turkey, where resilience was identified as a mediator in the relationship between fear of COVID and mental health outcomes (anxious and depressive symptoms) in university students (61). In Brazil, resilience had a mediating effect (B=0.14; 95% CI: 0.027-0.254) and explained 35% of the relationship between fear of COVID and anxiety (62). Similar results were reported by Javier-Aliega et al. in young individuals from Lima, where resilience was negatively correlated with fear of COVID-19 (B=-0.372, Beta= −0.251, TOL=0.594, VIF=1.675) (63).

Although we did not find a similar study that associated these variables in the military population studied in the context of the COVID-19 pandemic, there is literature that supports resilience as a protective factor against mental health problems, in addition to being associated with better military performance (64, 65). In addition, there is evidence suggesting that individuals with more resilience tend to externalize less fear and anxiety, which may be explained by the military training that rewards resistance, self-sufficiency and privacy, stigmatizing both physical and emotional weakness (66).

### Factors associated with fear of COVID-19

In our research, we found that the older the age, the higher the prevalence of fear of COVID-19. This is similar to what was reported in the United States by Niño et. al, who conducted a study in 10,368 American citizens. These authors identified that the older the age, the greater the perception of threat and, hence, the greater the fear of COVID-19 (30). This finding could be explained by the fact that these individuals are part of the population at risk of experiencing a more serious and complicated disease, with higher mortality rates (67–69). However, our findings align with those of Soraci et al. in the Italian population and Mistry SK et al. in Bangladesh, as we observed a significant association between age and fear of COVID-19 in our study (11, 12). On the contrary, Andrade et al, in Brazil, reported that the older the age, the lower the fear of COVID-19. The authors stated that the older population tends to worry less about death, has less knowledge of the disease and is generally more reluctant to accept safety measures (70).

Participants who identified as religious exhibited a 105% higher prevalence of fear of COVID-19. This finding contrasts with Prazeres et al.’s study in Portugal, which found no association between religiosity and fear of COVID-19 (36). In contrast, Ghoncheh K. et al. in Iran reported that individuals with greater religiosity and spirituality experienced less fear of COVID-19 due to their coping mechanisms rooted in their belief systems, helping them manage various difficulties and reduce psychological distress (37). The discrepancy in our study’s findings could be attributed to the workload of military and police forces, coupled with social distancing measures that restricted religious community gatherings, depriving individuals of the support typically derived from religious activities such as masses and spiritual retreats. This interpretation aligns with the observations made in the United States by Gomez et al., who analyzed the emotional impact of COVID-19 quarantine on individuals’ religiosity (38).

Overweight participants exhibited a 42% reduction in the prevalence of fear of COVID-19. While no similar studies were found, this finding contrasts with Chen et al.’s report in China, where overweight individuals showed higher levels of fear of COVID-19 compared to those with a normal BMI (71). The discrepancy might be attributed to sociodemographic and cultural factors (72–74). In our country and Latin America, being overweight might not be perceived as a health concern, as it is sometimes associated with strength and protection against adverse situations (75, 76), including the COVID-19 pandemic, despite the numerous studies linking overweight and obesity to higher morbidity and mortality from the disease.

The prevalence of fear of COVID-19 increases by 113% for participants who have a family member with a history of mental health issues. While no similar studies have been identified, this finding might be elucidated by existing evidence indicating that caregivers of individuals with mental illnesses experience significant physical and emotional stress. Consequently, family members of those with mental health issues may have a lower quality of life and a predisposition to conditions such as anxiety, depression, and post-traumatic stress disorder. These mental health challenges could potentially justify their heightened fear in the context of the COVID-19 pandemic (40, 41, 77).

The prevalence of fear of COVID-19 increases by 195% for participants with an eating disorder. This aligns with findings reported by Bemanian et al. in Norway and Dos Santos et al. in Brazil, both identifying a robust association between psychological stress related to COVID-19 and various eating disorders, including emotional eating and binge eating disorder (39, 78). Research supports the notion that eating disorders may serve as mechanisms for managing negative emotions through the release of serotonin and dopamine resulting from inadequate carbohydrate and lipid intake (79, 80). Additionally, restrictive eating patterns may be influenced by fear of COVID-19, including concerns about food quality and the potential for transmission (81). However, it’s worth noting that these findings contrast with studies by Ilktac et al. and Pak et al., who observed minimal associations between these variables. In their multiple models, the effect of eating disorders was diluted when considered alongside other confounding variables (82, 83).

### Implication of findings in mental health

Considering the great impact of the COVID-19 pandemic on mental health in the general population, the findings reported in this study support the need for the implementation of strategies focused on populations facing unique challenges, such as military personnel. Measures to address this situation could be taken through the strengthening of the Community Mental Health Centers, which should engage and establish an ongoing assessment of this population to avoid adverse consequences on their mental health.

The study’s findings underscore the significant impact of the COVID-19 pandemic on mental health, particularly among military personnel. To address the unique challenges faced by this population, future research and interventions should consider several key recommendations. Firstly, there is a need for the development and implementation of tailored mental health strategies specifically designed for military personnel. These strategies should take into account the distinct stressors and challenges associated with their roles during pandemic responses. Additionally, strengthening Community Mental Health Centers is crucial. These centers should actively engage with military personnel, conducting ongoing assessments to identify mental health concerns promptly. This proactive approach can help in avoiding adverse consequences on the mental health of military personnel. Exploring and implementing preventive measures is also important. Proactive approaches may include early intervention programs, resilience-building initiatives, and mental health education tailored to the unique needs of military personnel. Collaboration between mental health professionals, military authorities, and researchers is encouraged. Interdisciplinary approaches can provide comprehensive insights and facilitate the development of effective mental health interventions. Furthermore, conducting longitudinal studies to track the long-term mental health effects on military personnel is essential. Considering the evolving nature of the pandemic and its aftermath, these studies can provide valuable insights into the persistence and trajectories of mental health challenges. Extending research to include global comparisons is another recommendation. Examining how different countries and military structures cope with mental health challenges during and after pandemics through comparative studies can offer a broader perspective on effective strategies.

### Unique contributions to knowledge from a psychiatric perspective

In our primary investigation, we conducted a comprehensive cross-sectional study aiming to determine the prevalence and factors associated with depression and anxiety in Peruvian military personnel during the second wave of the COVID-19 pandemic (42). Our findings revealed a 29.9% prevalence of depression and a 22.0% prevalence of anxiety symptoms (42). On the other hand, distinctively contributing to our understanding of mental health in military contexts during the COVID-19 pandemic, our secondary analysis focuses on the fear of COVID-19 among military personnel. This exploration is vital, considering the unique challenges these individuals face, such as constant exposure to stressful situations and the pressure associated with being at the forefront of a health emergency response. The study not only unravels the prevalence of fear but also identifies specific factors influencing the mental well-being of military personnel. While the primary study provided a comprehensive exploration of depression and anxiety, the secondary analysis specifically hones in on the fear of COVID-19 within the military population. Together, these studies offer a nuanced understanding of mental health challenges, providing a more holistic view of the multifaceted psychological impact experienced by military personnel during the ongoing pandemic.

### Limitations and strengths

This study has several limitations. Firstly, the cross-sectional design hinders the attribution of causality among the variables under investigation. Secondly, there is a potential selection bias, as the primary study only encompassed one department, overlooking a significant portion of military personnel across other departments in the country. Nonetheless, a notable strength lies in the extensive probability sample obtained, and the instruments employed have been validated within our country. Furthermore, the study introduces novel variables, such as religion, eating disorders, and family history of mental problems, which were not previously considered in similar investigations.

Thirdly, we considered the potential for non-response bias. However, the study’s design, which involved gathering military personnel in groups and distributing the questionnaire within these controlled environments, minimized the likelihood of extensive non-response bias. The survey wasn’t widely promoted on social media platforms among the military; instead, participants were grouped and provided with the questionnaire link, ensuring a consistent response. Additionally, the key characteristics and sociodemographic features captured in our study align with known profiles of military personnel. This alignment is reflected in the 64.02% response rate, with only 10 military personnel declining to participate, indicating a generally representative sample. Fourth, there’s a potential for late bias as data collection occurred during the second wave of the COVID-19 pandemic, particularly in a phase of decreasing infection rates. However, during this period, Peru maintained significant measures such as mandatory social distancing, low vaccination coverage, and targeted quarantines in districts with high positivity rates. The reported 14.6% positivity and 14% lethality during epidemiological week 39 (the week when data was collected) in the Lambayeque region further highlights the ongoing impact of the pandemic during our study period, particularly on mental health and fear of COVID-19 among frontline populations such as military personnel (84). Fifth, there’s the potential for common method bias. However, we mitigated this risk by employing a diversified data collection approach, incorporating both in-person administration and electronic surveys. The instruments and questionnaires used in the study were meticulously designed and validated to ensure a comprehensive understanding of the constructs of interest (85, 86). Furthermore, we implemented various strategies, including unique identifiers, randomized question orders, and skip patterns in the electronic surveys, to enhance the reliability and validity of the data.

## Conclusions

In our conclusion, our findings highlight that a significant proportion, specifically two out of every ten military personnel, experienced fear of COVID-19. It is imperative to underscore the importance of directing focused attention towards the factors linked to the development of this fear within military personnel. Notably, age, religion, being overweight, having a family member with a history of mental illness, and having an eating disorder emerge as key determinants.

Acknowledging the inherent limitations of our study, we believe these results hold valuable insights for informed decision-making. They could significantly contribute to the formulation of effective mental health policies and targeted interventions across all levels of care. It is crucial to emphasize the need for considering military personnel as a priority group in the broader context of the post-pandemic scenario and future health emergencies or disasters. These considerations underscore the nuanced complexities of the mental health landscape, warranting tailored strategies for this particular population.

## Data availability statement

The dataset generated and analyzed during the current study is not publicly available because the ethics committee has not provided permission/authorization to publicly share the data, but it is available from the corresponding author upon reasonable request.

## Ethics statement

The studies involving humans were approved by Ethics Committee of Universidad San Martín de Porres (USMP) with the code 269-2022-CIEI-FMH-USMP. The studies were conducted in accordance with the local legislation and institutional requirements. The participants provided their written informed consent to participate in this study.

## Author contributions

DV-G: Conceptualization, Formal analysis, Investigation, Methodology, Project administration, Resources, Software, Supervision, Validation, Visualization, Writing – original draft, Writing – review & editing. HD-T: Conceptualization, Investigation, Methodology, Project administration, Resources, Software, Supervision, Validation, Visualization, Writing – original draft, Writing – review & editing. CP-R: Investigation, Methodology, Project administration, Resources, Software, Supervision, Validation, Visualization, Writing – original draft, Writing – review & editing. CV-M: Data curation, Investigation, Methodology, Project administration, Resources, Software, Supervision, Validation, Visualization, Writing – original draft, Writing – review & editing. VV-P: Conceptualization, Data curation, Formal analysis, Investigation, Methodology, Project administration, Resources, Software, Supervision, Validation, Visualization, Writing – original draft, Writing – review & editing. VF-R: Conceptualization, Investigation, Methodology, Project administration, Resources, Software, Supervision, Validation, Visualization, Writing – original draft, Writing – review & editing. CP-V: Conceptualization, Formal analysis, Investigation, Methodology, Project administration, Resources, Software, Supervision, Validation, Visualization, Writing – original draft, Writing – review & editing. DF: Data curation, Investigation, Methodology, Project administration, Resources, Software, Supervision, Validation, Visualization, Writing – original draft, Writing – review & editing. MV-G: Conceptualization, Data curation, Formal analysis, Investigation, Methodology, Project administration, Resources, Software, Supervision, Validation, Visualization, Writing – original draft, Writing – review & editing.
